# Supplementary material for: A toolbox of anti–mouse and anti–rabbit IgG secondary nanobodies
Source: J Cell Biol. 2018 Mar 5;217(3):1143–54. doi: 10.1083/jcb.201709115 (PMC5839796; doi:10.1083/jcb.201709115)
Supplement: Supplemental Materials [file JCB_201709115_sm.pdf]

Pleiner et al., https://doi.org/10.1083/jcb.201709115



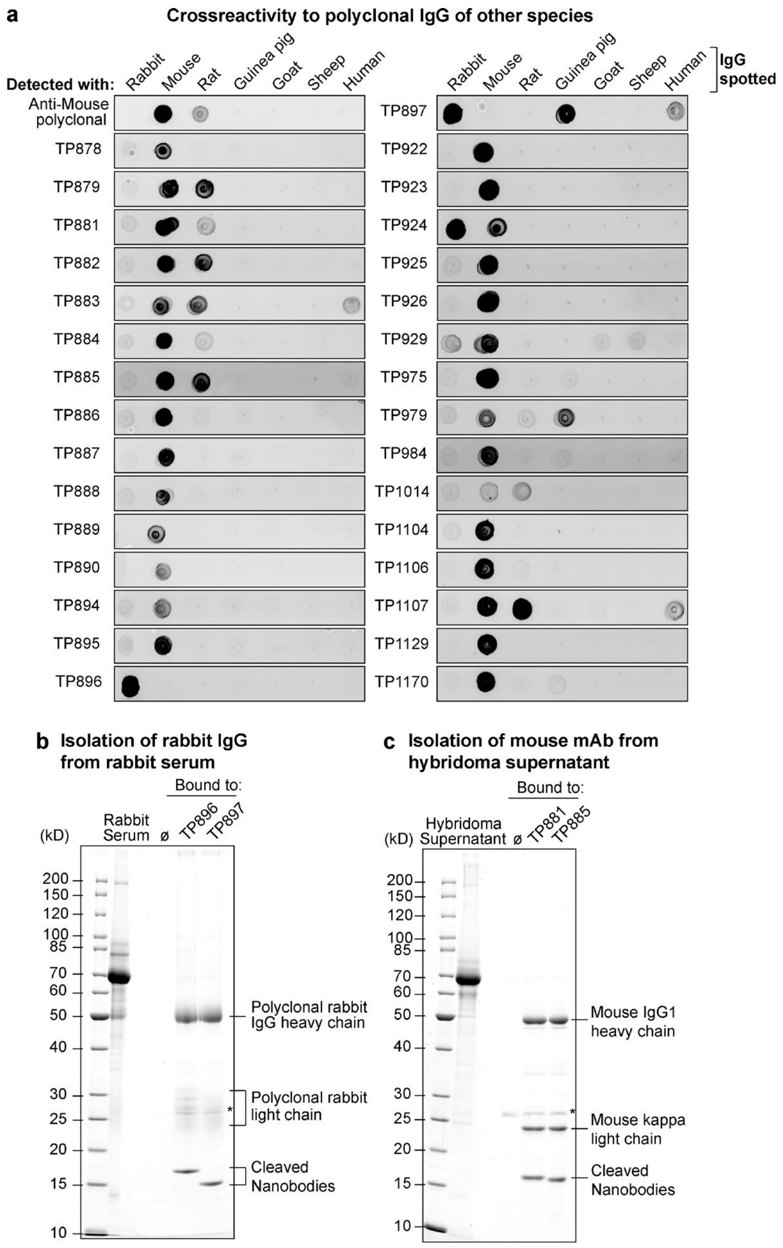


Figure S1. **Species cross reactivity profiling and native target IgG isolation. (a)** Cross reactivity profiling of all anti-IgG nanobodies. Using the same dot blot assay as described in Fig. 1 b, the cross reactivity of anti-IgG nanobodies to polyclonal IgG from the indicated species was determined. **(b)** Isolation of polyclonal rabbit IgG from rabbit serum. Anti–rabbit IgG nanobodies TP896 and TP897 carrying an N-terminal Avi-SUMOStar tag were biotinylated and immobilized on magnetic Streptavidin beads. After incubation with crude rabbit serum and washing, nanobody-bound polyclonal rabbit IgG was specifically eluted via SUMOStar protease cleavage in physiological buffer. Empty beads served as negative control. **(c)** Isolation of anti-Nup62 mouse IgG1 κ mAb A225 from hybridoma supernatant with anti–mouse IgG1 nanobodies TP881 and TP885 as described in panel b. The asterisk indicates the SUMOStar protease used for elution.

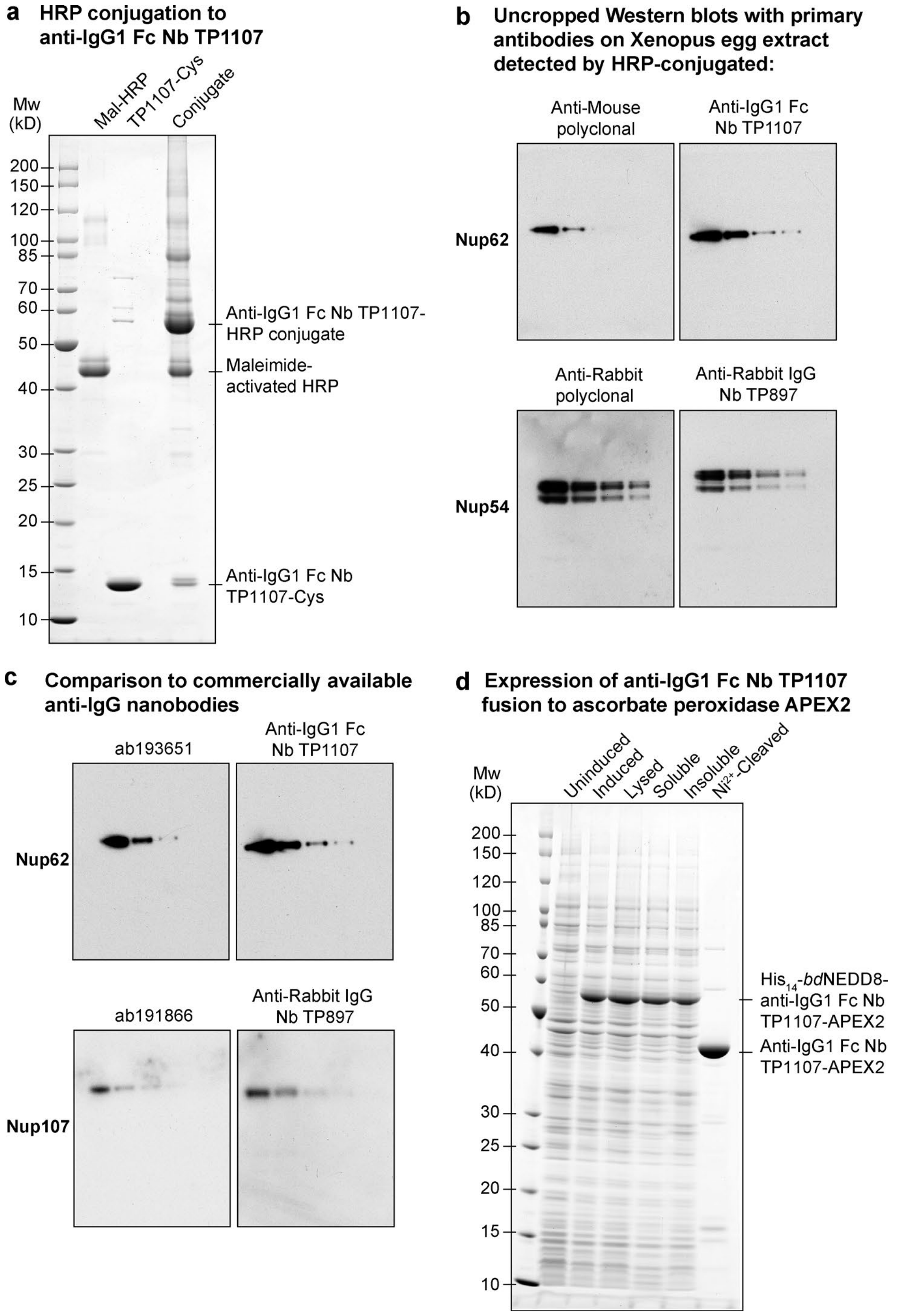


Figure S2. **Anti-IgG nanobody conjugation to HRP and fusion to APEX2. (a)** Anti-mouse IgG1 Fc nanobody TP1107 with a C-terminal cysteine was conjugated to maleimide-activated HRP by incubation of equimolar amounts for 1 h at room temperature. **(b)** Uncropped images of the Western blots shown in Fig. 2 a. Samples used for Western blots to compare the performance of polyclonal secondary antibodies and anti-IgG nanobodies were run on the same gel and blotted at the same time. The blots for a given primary antibody were developed under identical conditions in parallel. **(c)** A twofold dilution series of *Xenopus* egg extract was blotted on nitrocellulose and probed with an anti-Nup62 mouse IgG1 monoclonal antibody (top). It was then detected either via HRP-conjugated anti–mouse IgG1 nanobody ab193651 (1:3,500 dilution, ~5 nM; Abcam) or 5 nM HRP-anti–mouse IgG1 Fc nanobody TP1107. Bottom: A twofold dilution series of *Xenopus* egg extract was blotted on nitrocellulose and probed with a polyclonal rabbit antibody against Nup107. It was then detected either via HRP-conjugated anti–rabbit IgG nanobody ab191866 (1:3,500 dilution, ~5 nM; Abcam) or 5 nM HRP-anti–rabbit IgG nanobody TP897. **(d)** Expression of anti–mouse IgG1 Fc nanobody TP1107–APEX2 fusion in *E. coli*. After binding to nickel beads via the N-terminal $His_{14}$-*bd*NEDD8-tag, untagged fusion protein was eluted by on-column *bd*NEDP1 cleavage (Frey and Görlich, 2014).

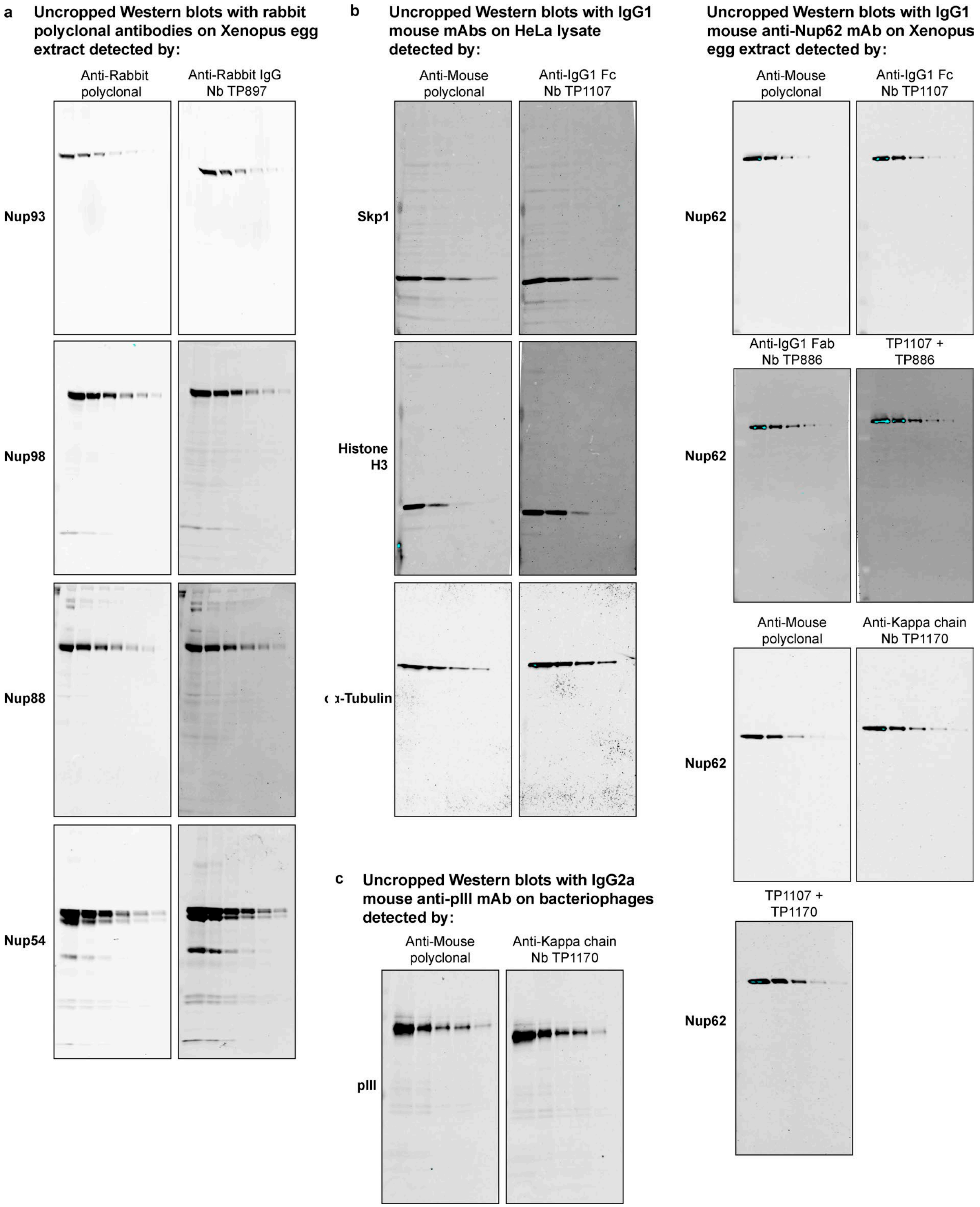


Figure S3. **Uncropped scans of the Western blots shown in Fig. 3. (a–c)** Samples used for Western blots to compare the performance of polyclonal secondary antibodies and anti-IgG nanobodies were run on the same gel and blotted at the same time. The blots for a given primary antibody were developed and scanned with identical settings in parallel. The molecular masses of the detected proteins are as follows: for each Nup, the molecular mass is part of its name; Skp1, 18.3 kD; Histone H3, 15.4 kD; α-tubulin, 50.1 kD; bacteriophage minor coat protein III, 44.7 kD.

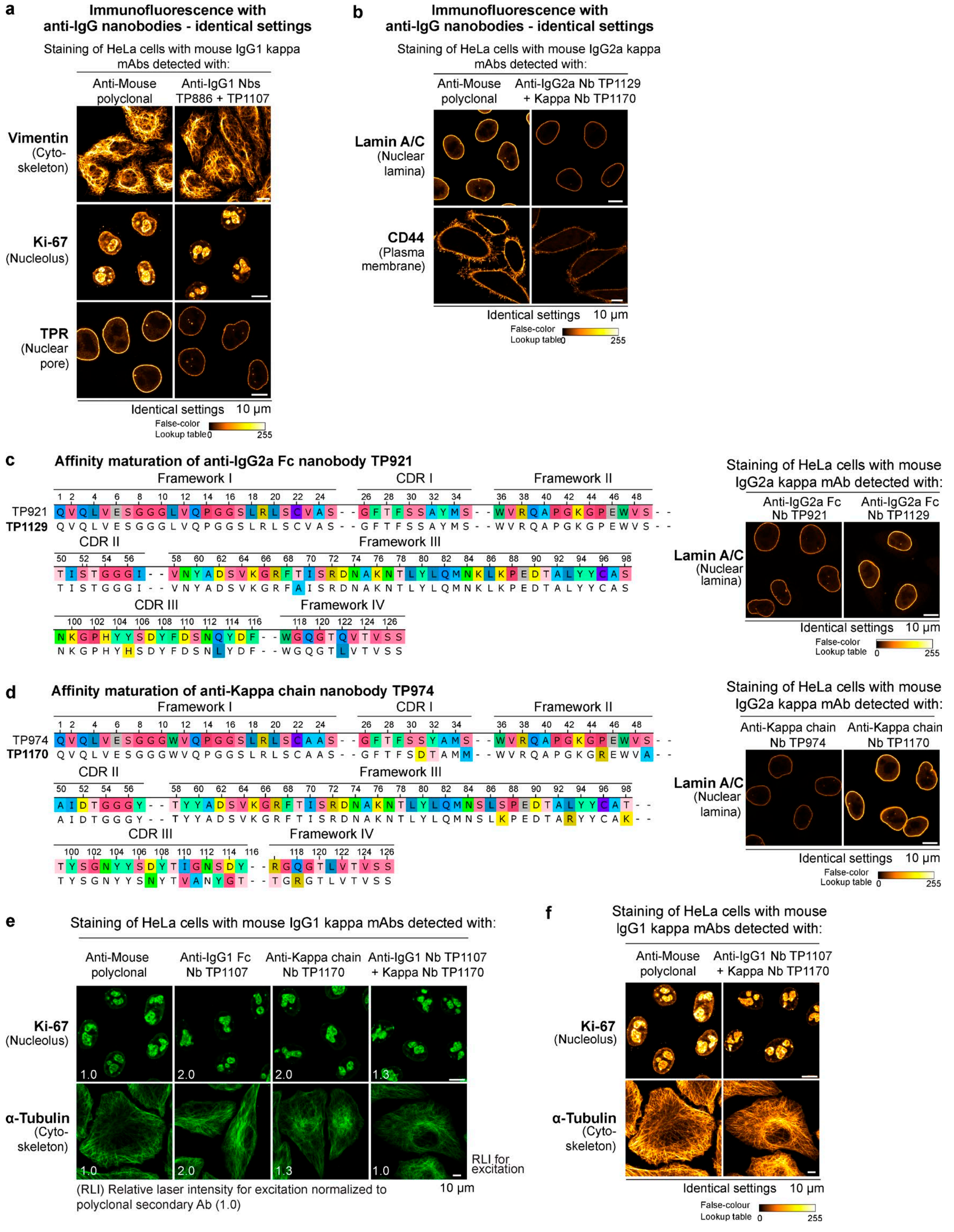


Figure S4. **Immunofluorescence with anti–mouse IgG nanobodies. (a and b)** Images for a given mAb or polyclonal antibody were acquired under identical settings, and pixel intensities are represented via a false-color lookup table. **(a)** HeLa cells were stained with the indicated mouse IgG1 mAbs. These primary antibodies were then detected with Alexa Fluor 488–labeled goat anti-mouse polyclonal antibody or a combination of anti–mouse IgG1 Fab nanobody TP886 and anti–mouse IgG1 Fc nanobody TP1107. **(b)** HeLa cells were stained with the indicated mouse IgG2a mAbs. These primary antibodies were then detected with Alexa Fluor 488–labeled goat anti-mouse polyclonal antibody or a combination of anti–mouse IgG2a Fc nanobody TP1129 and anti–κ chain nanobody TP1170. **(c)** Protein sequence alignment of anti–mouse IgG2a nanobody TP921 and the variant TP1129 obtained after affinity maturation. HeLa cells were stained with a mouse IgG2a mAb targeting Lamin A/C. The mAb was detected via TP921 or TP1129 labeled with a single Alexa Fluor 488 dye, and the images were acquired under identical settings. **(d)** Protein sequence alignment of anti–mouse κ chain nanobody TP974 and the variant TP1170 obtained after DNA shuffling and affinity maturation. HeLa cells were stained with a mouse IgG2a mAb targeting Lamin A/C. The mAb was detected via TP974 or TP1170, both labeled with two Alexa Fluor 488 dyes. **(e)** HeLa cells were stained with the indicated mouse IgG1 κ mAbs. These primary antibodies were then detected with Alexa Fluor 647–labeled goat anti-mouse polyclonal antibody, anti–mouse IgG1 Fc nanobody TP1107, or anti–mouse κ chain nanobody TP1170. A combination of TP1107 and TP1170 yielded increased staining intensities; see panel f for identical settings scan. RLI, relative laser intensity (as defined in Fig. 4 A).

**Provided online is Table S1 in Excel, which lists anti-IgG nanobody protein sequences.**

## Reference

Frey, S., and D. Görlich. 2014. A new set of highly efficient, tag-cleaving proteases for purifying recombinant proteins. *J. Chromatogr. A.* 1337:95–105. https://doi.org/10.1016/j.chroma.2014.02.029
